# A versatile framework for attitude tuning of beamlines at light source facilities

**DOI:** 10.1107/S1600577525003960

**Published:** 2025-06-12

**Authors:** Peng-Cheng Li, Xiao-Xue Bi, Zhen Zhang, Xiao-Bao Deng, Chun Li, Li-Wen Wang, Gong-Fa Liu, Yi Zhang, Ai-Yu Zhou, Yu Liu

**Affiliations:** a National Synchrotron Radiation Laboratory University of Science and Technology of China Hefei Anhui230029 People’s Republic of China; b Institute of High Energy Physics Chinese Academy of Sciences Beijing100049 People’s Republic of China; c University of Chinese Academy of Sciences Beijing100049 People’s Republic of China; RIKEN SPring-8 Center, Japan

**Keywords:** beam focusing, sample alignment, *Mamba*, virtual beamlines, software architecture

## Abstract

Aside from regular beamline experiments at light sources, the preparation steps before these experiments are also worthy of systematic consideration in terms of automation; a representative category in these steps is attitude tuning, which typically appears in contexts like beam focusing, sample alignment *etc*. With the goal of saving time and human effort in both writing and using such code, a *Mamba*-based attitude-tuning framework is created. It supports flexible input/output ports, easy integration of diverse evaluation functions and free selection of optimization algorithms. With the help of *Mamba*’s infrastructure, machine learning (ML) and artificial intelligence (AI) technologies can also be readily integrated. The tuning of a polycapillary lens and of an X-ray emission spectrometer are given as examples for the general use of this framework, featuring powerful command-line interfaces (CLIs) and friendly graphical user interfaces (GUIs) that allow comfortable human-in-the-loop control. The tuning of a Raman spectrometer demonstrates more specialized use of the framework with customized optimization algorithms. With similar applications in mind, this framework is estimated to be capable of fulfilling most attitude-tuning needs. Also reported is a virtual-beamline mechanism based on easily customisable simulated detectors and motors, which facilitates both testing for developers and training for users, as well as the encapsulation of digital twins.

## Abstract 2

A versatile *Mamba*-based software framework for automated attitude tuning of beamlines is reported, which is expected to be able to cover most attitude-tuning needs (beam focusing, sample alignment *etc.*) in a simple and maintainable way. As well as a few real-world examples at the High Energy Photon Source and Beijing Synchrotron Radiation Facility, both in China, also presented is a virtual-beamline mechanism based on easily customisable simulated detectors and motors.

## 1. Introduction

In beamline experiments, apart from the main scan steps (‘counting’, step scans or fly scans, and also including their data processing), the preparation steps before them can also be of considerable complexity, and therefore be of particular interest in terms of automation. Representative categories in these steps are beam focusing (Hong *et al.*, 2021 ▸; Xi *et al.*, 2017 ▸) and sample alignment (Robertson *et al.*, 2015 ▸; Zhang *et al.*, 2023*a* ▸) *etc*. At HEPS, the High Energy Photon Source (Xu *et al.*, 2023 ▸), and BSRF, the Beijing Synchrotron Radiation Facility, we refer to these steps as *attitude tuning*, borrowing the term ‘attitude’ from aerospace engineering (the term ‘configuration’ could also be considered, but would be very ambiguous). In our eyes, the essence of attitude tuning is the optimization of certain objective parameter(s) by manual or automated tuning of the corresponding attitude parameters: *e.g.* the automatic focusing of cameras is the automatic optimization of some image definition functions by tuning parameters like focal lengths. From the information we have collected from BSRF and HEPS and our colleagues’ visits to other facilities, we find attitude tuning a ubiquitous requirement at these facilities. Given this background, we find that most of them are essentially peak finding, albeit often in >1D configuration spaces.

As HEPS and other advanced light sources have small light spots and high brightness, traditional scan-based methods for attitude tuning could not only waste a lot of time (especially in >1D tuning applications; see Section 3 for one example), but also potentially result in more radiation damage to samples. Also, considering that attitude-tuning requirements need to be implemented for the 15 beamlines in Phase I of HEPS, it is imperative for us to create an efficient and unified software framework for attitude tuning. Fortunately, *Mamba* (Liu *et al.*, 2022 ▸; Dong *et al.*, 2022 ▸), the *Bluesky*-based (Allan *et al.*, 2019 ▸) software environment created for beamline experiments at HEPS, was also designed with attitude tuning and other preparation steps in mind from the very beginning. Based on *Mamba*, we have created a versatile framework for attitude tuning, where peak finding can be done in a simple, consistent and maintainable way. The code for our framework is available as part of the open-source edition of *Mamba* at https://codeberg.org/CasperVector/mamba-ose; in the event of future changes, this paper refers to version 0.4.4 of it at https://codeberg.org/CasperVector/mamba-ose/src/commit/47698067.

Although conceptually simple, beamline attitude tuning can often be complex in practice. While often separable from the main scan steps and therefore operated by beamline staff, attitude tuning can also become intertwined with the experiments and may need to be done by regular users. Camera-assisted automated sample changing can be regarded as an example of user-oriented tuning; to us, the main challenge with this kind of tuning is the necessity of a friendly graphical user interface (GUI). Many kinds of devices can be involved, which can again vary greatly across different applications. As well as diverse types of detectors and motors, compound or special devices like monochromators and robots may also be used; another major type of device that has recently seen rapid growth in use is digital twins (Feuer-Forson *et al.*, 2024 ▸; Whittle *et al.*, 2022 ▸). In many cases, the objective parameters are obtained in straightforward ways, *e.g.* by counting 0D/1D/2D detectors and perhaps applying region-of-interest (ROI) processing. Sometimes the processing can be more complicated, and sometimes the objective value after each move may even need to be obtained by a scan of some sort (ptychography, tomography *etc.*). For light sources that can produce small light spots, attitude tuning based on ptychographic wavefront measurement (Takeo *et al.*, 2020 ▸) is particularly crucial. Many optimization algorithms are available: single-objective or multi-objective, local or global, unbounded or bounded, gradient-free or gradient-based *etc*. With recent advances in machine learning (ML) and artificial intelligence (AI), their applications in attitude tuning, *e.g.* Bayesian optimization strategies (Morris *et al.*, 2024 ▸; Rebuffi *et al.*, 2023 ▸) and data processing pipelines empowered by deep learning (Zhang *et al.*, 2024 ▸), are also increasingly common. In Section 2, we will see how these complexities are dealt with in our attitude-tuning framework, while the cost of implementation in each case is kept close to the minimum.

## 2. Architecture of the attitude-tuning framework

**Figure 1:**
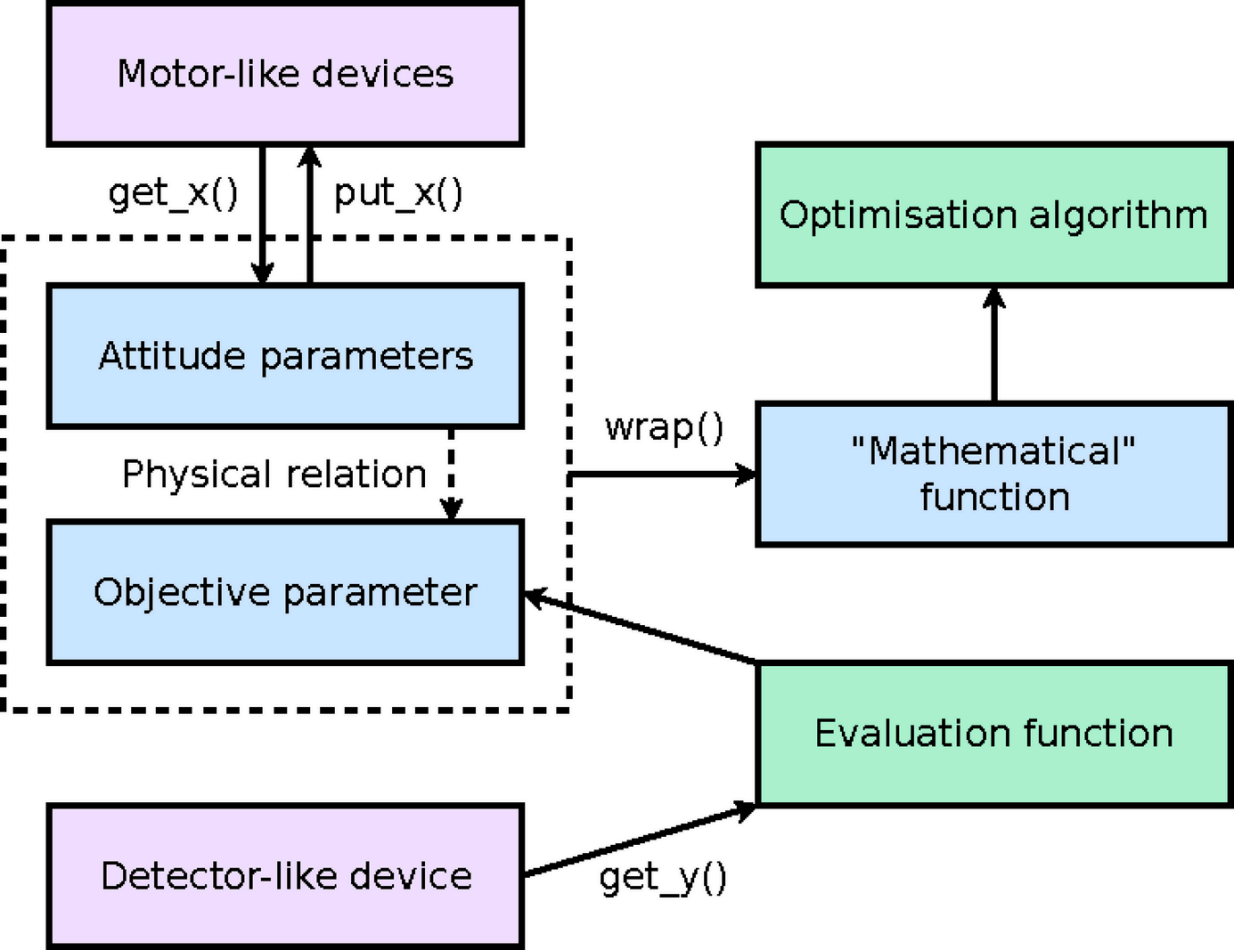
Architecture of the attitude-tuning framework based on AttiOptim.

**Figure 2:**
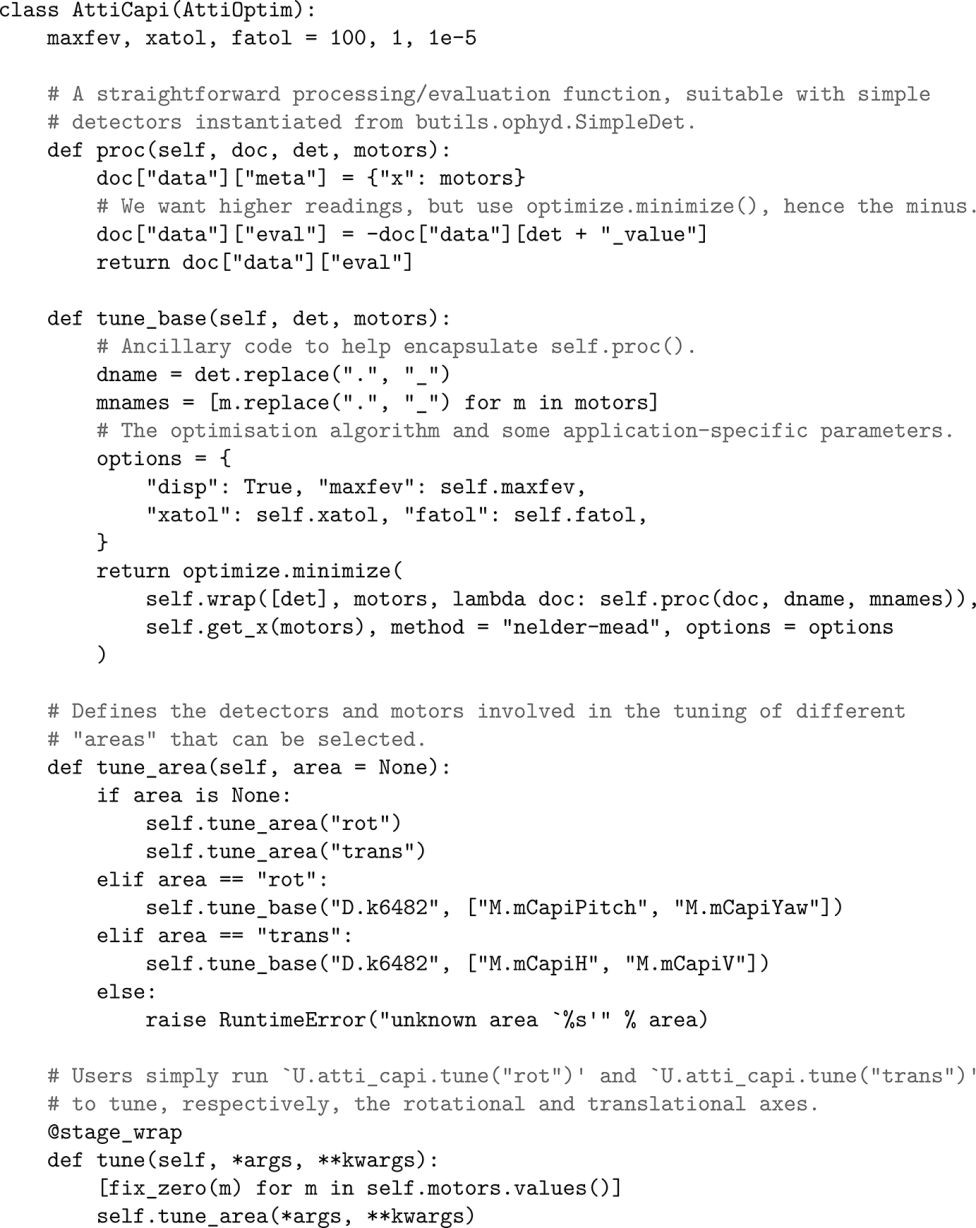
Notable fragments of 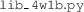 with brief usage notes.

As has been noted in Section 1, we treat attitude tuning as a matter of numerical optimization; therefore the architecture of a general-purpose attitude-tuning framework (Fig. 1 ▸) will inevitably include some attitude parameters, some objective parameter(s) and an optimization algorithm. Moreover, since the objective parameter(s) are actually obtained by physical measurement instead of purely mathematical computation, the architecture also needs to include detectors, motors and evaluation functions which convert the raw data from the detectors into objective values. Given these architectural elements, we implemented the 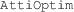 class which cooperates with *Bluesky*’s unified interfaces for motors and detectors, as well as optimization libraries like 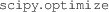. The functions 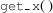, 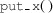 and 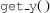 in this class deal with motors and detectors by interacting with their *Bluesky* encapsulations. The function 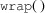 combines specified processing/evaluation functions with 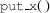 and 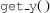 into functions with a seemingly purely mathematical signature, which are required by libraries like 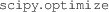 (Fig. 2 ▸).

In Section 3, we will see real-world applications of our framework, using 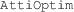, on a polycapillary lens and an X-ray emission spectrometer. The former is more general, with a straightforward processing/evaluation function; the latter is more specialized, and demonstrates the use of a much more complex evaluation function. Then, in Section 4, we will see an even more specialized application of our framework on a Raman spectrometer, where the ‘optimization algorithm’ interface (see also Section 5) is ‘abused’ to do parallelized peak finding of multiple objective parameters, as well as a task that is not even numerical optimization at all. By customizing 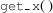 and 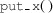, it is also possible to manipulate general motor-like devices, including monochromators and robots that expose motor-like interfaces. This is based on *Bluesky* encapsulation on top of *EPICS* IOCs and/or Python IOCs (Li *et al.*, 2024 ▸), or in a few cases direct *Bluesky* encapsulation of devices (Li *et al.*, 2023*a* ▸); in Section 4, a virtual-beamline mechanism will be introduced, which can also be used to encapsulate digital twins. With more advanced customization of 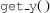 and 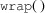, it is also possible to encapsulate procedures succinctly to obtain objective values from scans as ‘mathematical’ functions; one example for this will be covered in Section 5.

It can be seen from the above that our attitude-tuning framework handles the complexities resulting from the diversity of devices and the need sometimes to obtain objective values from scans by careful *modularization*. To put it another way, while 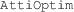 may appear simple, based on it our framework delegates the tasks in attitude tuning to other dedicated components: device encapsulation to *Bluesky*, scans (including their data pipelines) to *Mambaetc*. The same approach is followed for the other complexities noted in Section 1; in this way, our framework helps developers to compose building blocks in attitude tuning with maximum freedom yet minimal effort. We place no artificial restrictions on the optimization algorithms, as long as they are encapsulated under interfaces similar to 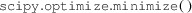. ML-based optimization can also be used, and ML/AI-based data pipelines can be integrated just like their regular counterparts. With the submit/notify pattern (Li *et al.*, 2024 ▸) based on the separation between backends and frontends, GUIs can be implemented with minimal amounts of code and degrees of coupling. In combination with minimal command-line interfaces (see Fig. 2 ▸ for one example) and implementations of the tuning logic behind them, they minimize the cost of attitude-tuning applications for both developers and users. From the information we have collected from HEPS, BSRF and other facilities, even with all the complexities above, among all the attitude-tuning requirements at these facilities, most are still peak finding in essence. For these reasons, we believe our framework is general enough to handle these kinds of application scenarios, saving great amounts of time and energy while only requiring very modest amounts of programming.

**Figure 3:**
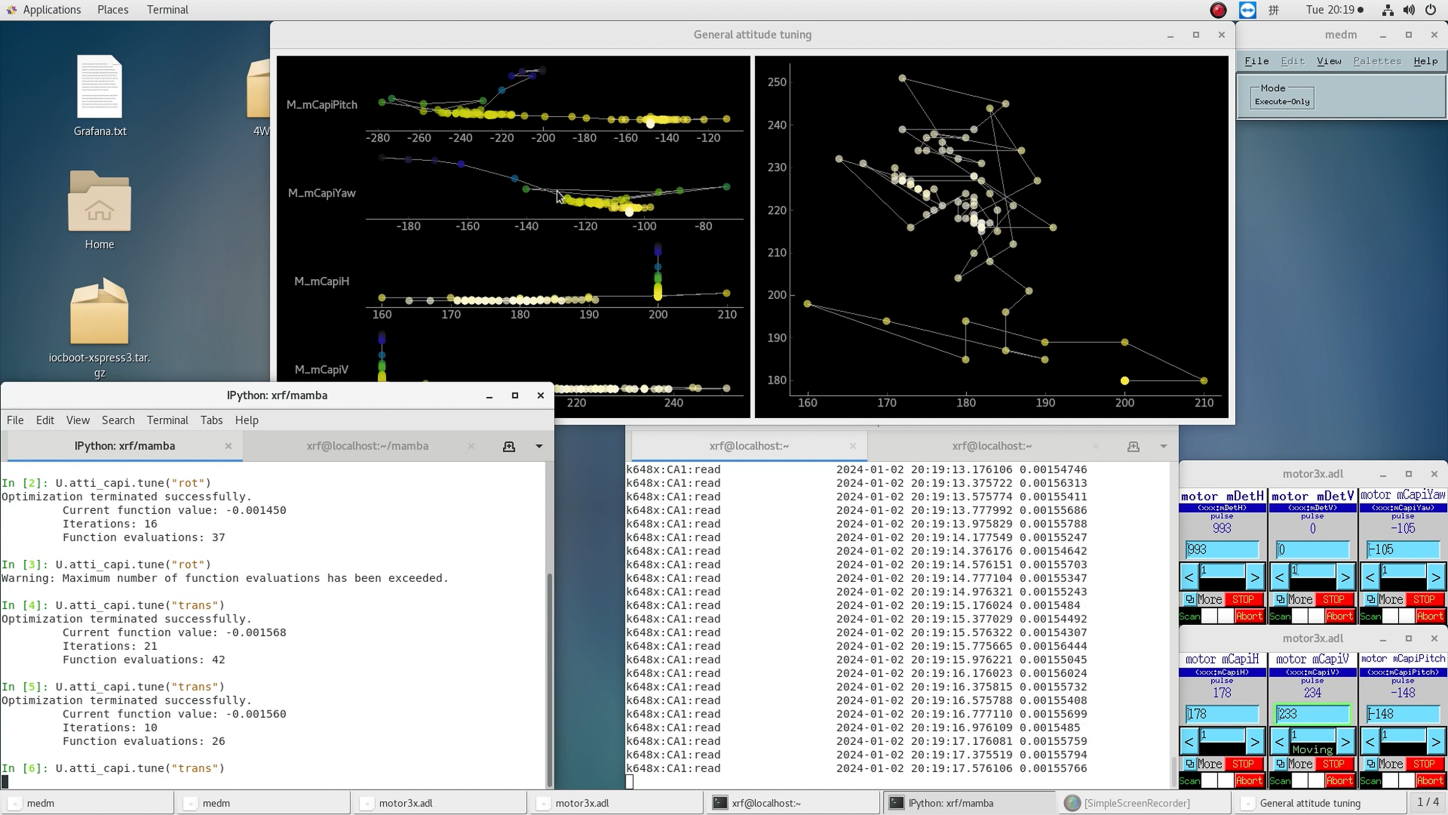
Attitude tuning of the polycapillary lens on 4W1B at BSRF. In the bottom left is a command-line backend window where commands provided by 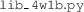 can be entered; other windows on the bottom monitor the detector readings and motor positions. At the top is the 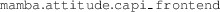 window, where 1D and 2D projections of the motion trajectories in the configuration space are monitored. In this window, brighter dots indicate better values for the evaluation function; in the 1D view in the left-hand pane, better values are also plotted with lower vertical coordinates. We also note that the convergence of the tuning procedure was decided by the operator, mainly depending on the detector readings after repeated runs, as the motors involved had no encoders and BEPC II was running in its decay mode.

Although our attitude-tuning framework supports free selection of optimization strategies, in our own applications based on it we still have a certain preference for some strategies, which is the subject of the rest of this section. In Section 3, we have chosen the local Nelder–Mead algorithm instead of *e.g.* the global ML-based Bayesian optimization, and a two-pass tuning strategy for the polycapillary lens instead of a one-pass strategy tuning all the axes, and we enforce limits on the numbers of optimization moves instead of solely relying on natural convergence of the algorithms [Figs. 3 ▸, 4 ▸ and 7(*a*)]. These are because we have learned from prior experience that the behaviours of the objective parameters are suitable for local optimization, and in the polycapillary case suitable for separate tuning of the two groups of axes, both of which help to save time. A limited financial budget results in the use of motors without encoders and a preference for simple resource-efficient algorithms. Both factors make the Nelder–Mead algorithm preferable in practice, and the former (together with the decaying beam current of BSRF, Section 3) also results in a lower total cost when letting the operator decide the convergence. The minimization of complexity/cost, or in other words approaching complexity lower bounds, is the main theme throughout our works. The observation by Graham (2002 ▸) about the succinctness of programming languages may be interpreted as a proposition that the amount of information a human can process in a fixed period of time is constant. This leads to the corollary that human intelligence coincides with the capability to minimize complexity/cost, which also corresponds neatly to Hutter’s (2020 ▸) treatment of AI as a matter of information compression. Therefore we advocate for the *collaboration and coevolution of AI with human intelligence*. With an AI, we would ultimately expect solutions similar to those above, given similar problems and constraints; as a first step, it should be able to recognize autonomously the suitability of local optimization and the separability between different groups of axes.

## 3. General attitude tuning: a polycapillary lens and an X-ray emission spectrometer

Our first example concerns the polycapillary lens on the 4W1B beamline at BSRF, which has four motorized degrees of freedom: a pitch/tilt angle (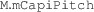), a yaw/pan angle (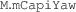), a horizontal shift (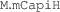) and a vertical shift (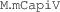). Based on prior experience, they are split into two groups; the rotational parameters are the coarse tuning parameters, and the translational parameters are the fine tuning parameters, where the former usually need to be tuned before the latter. The goodness of the lens’ attitude is determined by the readings from a Keithley 6482 picoammeter (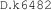), which is connected to a photodiode temporarily placed next to the lens when attitude tuning is performed. The code fragments used for this tuning based on our framework, 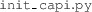 and 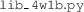, are available in the supporting information. The former mainly does *Bluesky* encapsulation of the devices involved, while the latter (with notable fragments and brief usage notes in Fig. 2 ▸) contains the tuning logic. Online visual feedback of the tuning procedure can be done with our general-purpose GUI for attitude tuning, 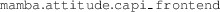 (Fig. 3 ▸). A more feature-complete version of the GUI is shown in Fig. 7(*a*), which most notably allows for the manual selection of axes (called *x* and *y* therein) for the 2D visualization in the right-hand pane; otherwise the GUI would automatically select the axes based on the latest data update.

Attitude tuning for the polycapillary lens on 4W1B at BSRF needs to be done roughly once per day during normal operation of the beamline, where it used to be done by manual trial and error, each time costing around half an hour. With our attitude-tuning framework, the procedure has been greatly accelerated and simplified, costing just a few minutes each time and demanding much less manual intervention. For historical reasons, scan-based tuning had not been used on the beamline, but it is pretty clear from Fig. 3 ▸ that this approach would be significantly less effective. Two-dimensional grid scans would be slow, while 1D scans would need quite a number of re-scans for convergence, considering that the latter are essentially a non-optimal iterative line-search strategy. Also adding to the complexity is the decaying beam current of the storage ring when the underlying facility, the Beijing Electron–Positron Collider II (BEPC II), is running in its decay mode. We are also aware of the potential need to tune multiple groups of attitude parameters, where not all groups are tuned according to the same objective parameter. In response, our framework is designed to be capable of *multi-objective tuning*. A simple example is given in the files 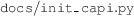 and 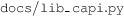 from the open-source edition of *Mamba*. In the general-purpose visualization GUI [Fig. 7(*a*)], the objective parameter to visualize can be selected from the *z*-axis menu.

**Figure 4:**
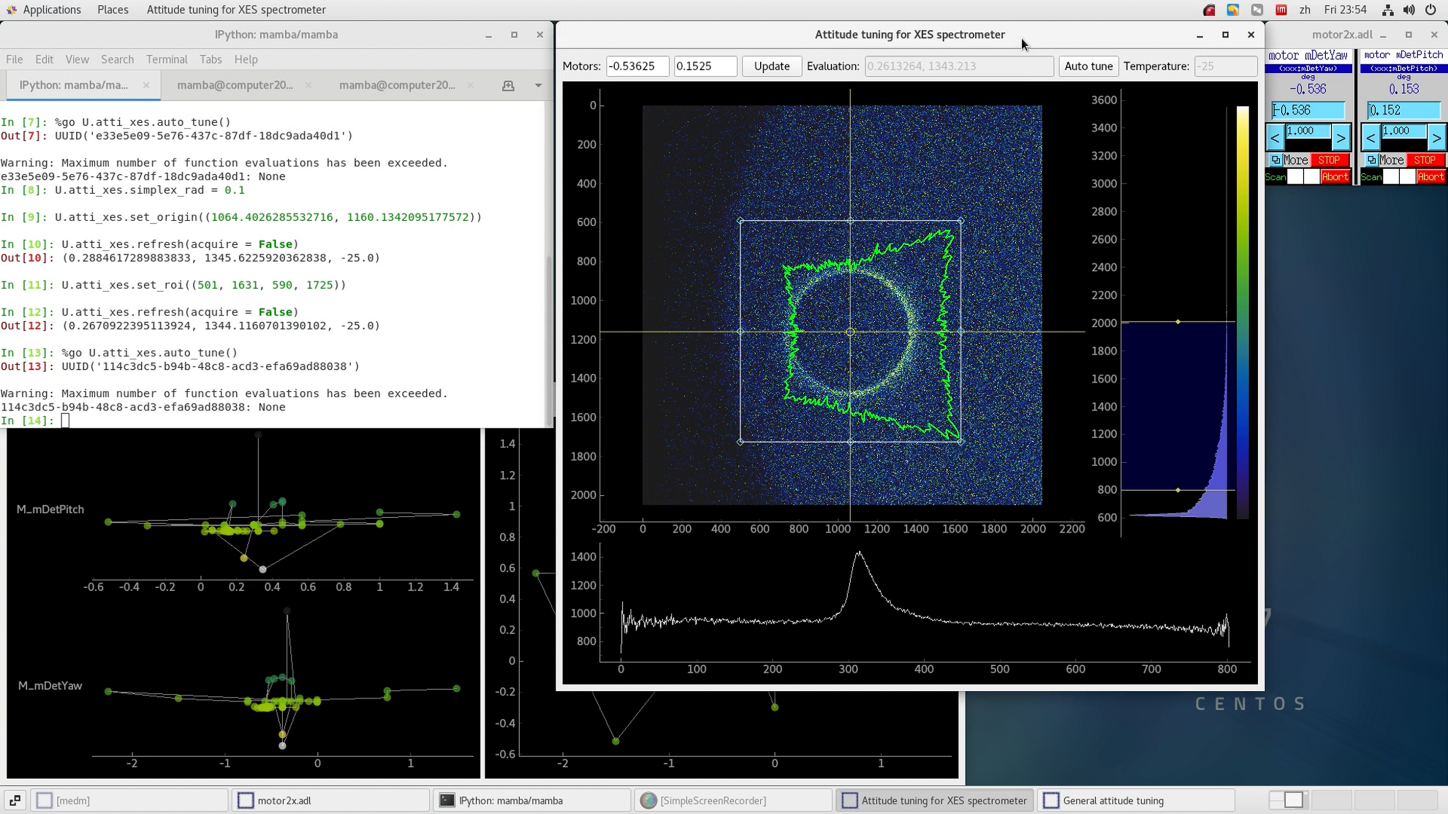
Attitude tuning of an XES spectrometer on 4W1B at BSRF. The main window shows the image acquired from the detector, where the circular pattern can be observed, and its shape needs to be optimized. Also shown are the radial (bottom) and angular (irregular curve in the top left) distributions computed from the image. The ROI and origin used in the computation are shown (and can be modified), respectively, with the rectangle and the crosshair in the top left.

Another example for our attitude-tuning framework relates to a full-cylindrical von Hamos spectrometer (Guo *et al.*, 2023 ▸) for X-ray emission spectroscopy (XES), currently used on 4W1B at BSRF. It is also planned to be used on the high pressure beamline (B6), the X-ray absorption spectroscopy beamline (B8) and possibly other beamlines at HEPS. Bragg reflection from the analyser of the spectrometer produces circular patterns in images acquired from the detector (Fig. 4 ▸). The pitch/tilt and yaw/pan angles are tuned to optimize the shape of the circle, so that the sharpest peak is obtained on the radial distribution curve shown on the lower pane of the main window in the figure. More precisely, the half-maximum region of interest (HM-ROI) is computed for the radial distribution, and then the mean intensity in this ROI is used as the objective parameter. From the description above it can already be seen that for the tuning of this spectrometer, the processing/evaluation function needed must have a non-trivial complexity. But in addition to this, another main source of complexity is the selection of the centre (origin) of the circles, where inappropriately selected origins can result in distorted peaks and non-optimal attitudes. According to actual tests, the simple barycentre algorithm often fails to find an optimal origin, and we currently use numerical optimization of the HM-ROI mean mentioned above over candidates for the origin.

Noting that automated origin selection is a computationally expensive procedure, which however only needs to be done after significant changes to the attitude, we decided to use a *human-in-the-loop* approach for it. An ‘Auto origin’ button is provided in the GUI [see the more feature-complete version in Fig. 7(*a*)] for automated fine tuning of the origin, whose coarse selection can be done manually by dragging the origin crosshair shown in the GUI. In the light of the analyses above, we wrote a specialized attitude-tuning program for this XES spectrometer as 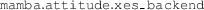 and 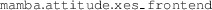, where the latter is the main GUI shown in Fig. 4 ▸. Considering that radial distributions and raw images are the main data wanted by users in normal data acquisition (‘counting’) after attitude tuning has been done, this program is also written with normal counting in mind. The GUI provides features desired by users, like setting the acquisition time and saving experiment data. XES attitude tuning is normally done by beamline staff, and the tuning procedure (except for origin selection) is already convenient enough for them on a command line, so there is no GUI button for it. Other than the general-purpose 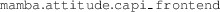 (as with the polycapillary lens above), visualizations in the specialized GUI will also be automatically updated after each move in the tuning procedure.

## 4. Specialized attitude tuning: an XRS spectrometer

**Figure 5:**
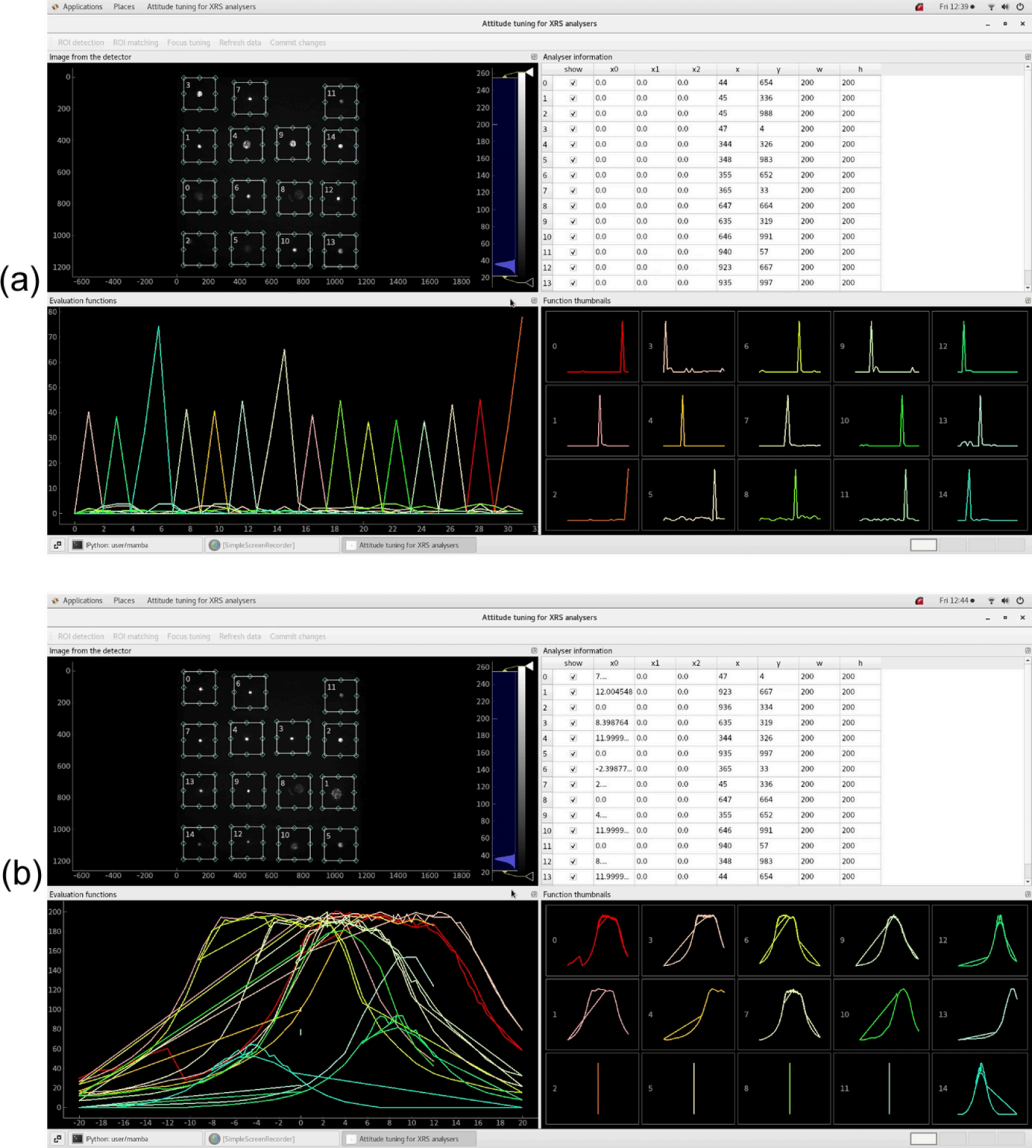
GUI of our XRS attitude-tuning program, doing (*a*) ROI matching and (*b*) focus tuning; the pictures are obtained from a laser-based simulation of what would eventually be done with X-rays at HEPS. Motor motion and ROI editing can be done in the top right-hand pane; ROIs can also be modified with mouse operations on ROI rectangles in the top left-hand pane, and/or with drag-and-drop operations of table rows in the top right-hand pane. Automated tuning is currently only implemented for a single analyser module, while parallelized tuning of multiple modules will be implemented in the future. Apart from a GUI with a quite different appearance, the latter would also require coordination between multiple threads responsible for the tuning of the analyser modules. This is because the spectrometer on B5 at HEPS uses Lambda Flex detectors, where multiple detector heads on the analyser modules belong to the same detector unit and are not triggered individually.

Among the instruments on the hard X-ray high-resolution spectroscopy beamline (B5) of HEPS, currently under active construction, is an X-ray Raman scattering (XRS) spectrometer. Structurally similar to the spectrometer discussed by Huotari *et al.* (2017 ▸), the XRS spectrometer on B5 at HEPS has six analyser modules, each containing a detector and a 3 × 5 array of analysers, where each analyser has three motorized degrees of freedom – one longitudinal shift and two latitudinal angles. As shown in Fig. 5 ▸, latitudinal tuning (*x*_1_ and *x*_2_ for each analyser) moves the X-ray spots around on the detectors, while longitudinal tuning (*x*_0_ for each analyser) focuses the spots. After the spots are properly distributed and each of them is correctly assigned to the corresponding analyser, focus tuning is performed. All these steps can be done in the GUI of our specialized attitude-tuning program for this spectrometer, 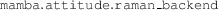 and 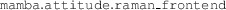. Spot distribution is done manually with visual aid from the GUI, spot assignment (ROI detection followed by ROI matching) is automated, and focus tuning is automated and parallelized. Due to the use of multiplexers for motion controllers (Li *et al.*, 2024 ▸), the motors in each analyser module are separated into multiple groups, where two motors in the same group cannot be moved at the same time. Consequently, in our program the parallelization of focus tuning is done in multiple passes: the first motor in every longitudinal group, then the second *etc*.

Focus tuning of the XRS spectrometer on B5 at HEPS is based on a 2D generalization of the HM-ROI mean in Section 3 as the objective parameter. Other than its application on spectrometers in this paper, similar evaluation functions have also been used in a few other scenarios, *e.g.* the standalone X-ray beam-position monitor (XBPM) program presented by Li *et al.* (2024 ▸) based on images from area detectors. The optimization is implemented by multiple calls to 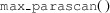, a function that optimizes, in parallel, the objective parameter for each ROI in the list of ROIs passed to this function. This function’s signature is similar to *e.g.*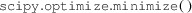, except that its objective function produces a 1D numerical array instead of a 0D number. Internally, it first does a coarse inner-product scan [*cf.*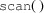 from *Bluesky*] for elements of the input parameter, then does a fine inner-product scan of the input elements in the reverse direction, and finally sets the input elements to the peak positions (see Fig. 9 for details). ROI matching is implemented with 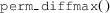, a function with a signature similar to 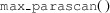; its ‘objective function’ computes all X-ray spots’ barycentres inside their HM-ROIs (which are also used in the XBPM program mentioned above), as well as the distance from each barycentre to a corresponding reference position. For each analyser, 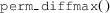 sets the reference positions to the initial positions of the barycentres. It then changes the latitudinal parameters of this analyser in small steps until a spot/ROI stands out with a significantly larger distance than all the rest. This outstanding ROI is assigned to the current analyser and the latitudinal parameters are reset to the original values. After that the reference positions are updated to counteract potential motor backlashes, and then 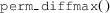 moves on to the next analyser. Finally, 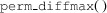 returns the mapping table from ROIs to analysers, which is applied in the command-line backend and GUI frontend of our program.

**Figure 6:**
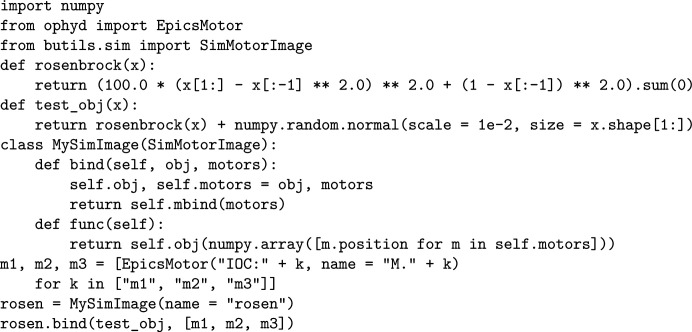
A virtual device connected to three *motorMotorSim*-based motors, based on the Rosenbrock function widely used to test optimization algorithms, perturbed by a random noise.

**Figure 7:**
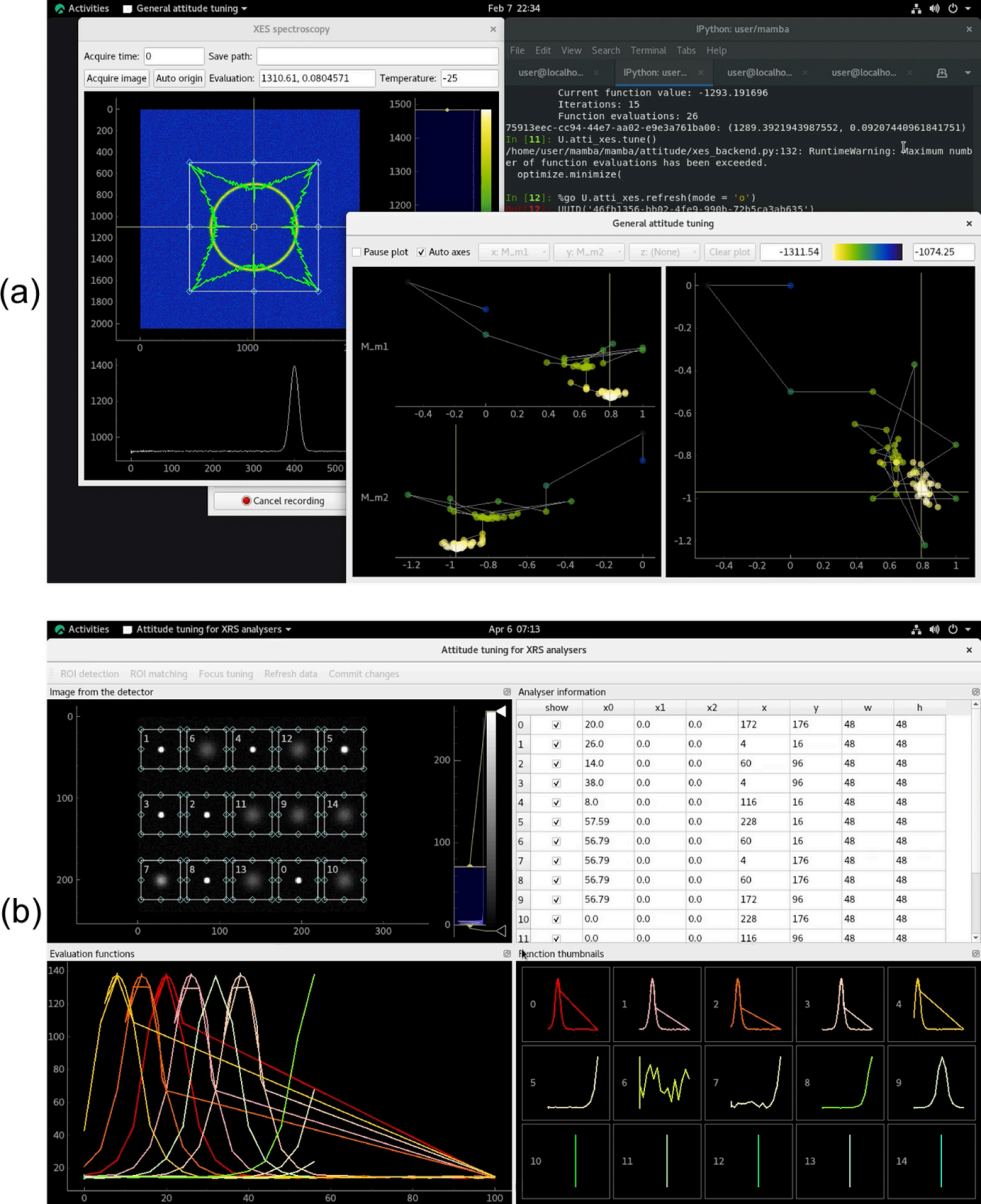
Our (*a*) XES and (*b*) XRS attitude-tuning programs running with virtual beamlines. In comparison with its counterpart in Fig. 4, the window in panel (*a*) on the left is extended with widgets to set the ‘counting’ parameters for regular users, and an ‘Auto origin’ button for automated fine tuning of the origin. In comparison with its counterpart in Fig. 3, the window in panel (*a*) at the bottom right is extended with widgets that set whether to pause plotting, as well as whether to select automatically which axes and objective parameter to visualize. When the former is enabled, the added ‘Clear plot’ button can be used. When the latter is disabled, the attitude parameters for the 2D projection in the right-hand pane can be selected from the *x*- and *y*-axis menu, while the objective parameter can be selected from the *z*-axis menu.

To finish this section, we note that while 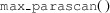 and 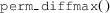 differ from 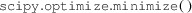, they are actually more like 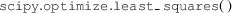 (see also Section 5). The latter may be seen as the simplest kind of optimization that considers multiple objective parameters simultaneously instead of successively, unlike the 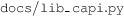 case in Section 3. This proves the capability of our attitude-tuning framework to do fully fledged multi-objective tuning (Zhang *et al.*, 2023*b* ▸; Rebuffi *et al.*, 2023 ▸), noting that it is easy to substitute least squares with other multi-objective algorithms. Here we also briefly introduce the *virtual-beamline mechanism* which we now use extensively, both in attitude tuning and in other applications. First, based on the *motorMotorSim* and *ADSimDetector* modules in *EPICS*, we are already able to perform many kinds of simulations, also including those independent of *Bluesky*, *e.g.* the Python IOC for motor multiplexers described by Li *et al.* (2024 ▸). We note that the former is especially useful because of its realistic simulation of motor speeds, soft limits *etc*. Second, we also created the 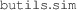 module for *Mamba*, which provides useful and easy-to-use simulation device classes that expose interfaces similar to those of *Bluesky*’s classes for real devices. For example, the 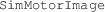 class (Fig. 6 ▸) implements a virtual device that binds to a simulation function and some motor-like device object(s), whether real motors, simulated motors like those based on *motorMotorSim*, or even things like the energies of monochromators; it produces readings according to the device positions and the simulation function. Based on the mechanisms above, we are able to create virtual beamlines to test programs and train both staff and users, saving much beamtime and allowing for the development of software before the required instruments are fully ready. For instance, with virtual beamlines we can test our attitude-tuning programs (Fig. 7 ▸; all the simulation code is available in the 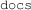 subdirectory of the open-source edition of *Mamba*) extensively before their tests on real hardware, and often only need minor tweaks/fixes in later tests. We also note that this mechanism may also be used to encapsulate digital twins; apart from simulations oriented towards testing and training, it may also facilitate information feedback from the optimization procedure (where real-world measurement takes place) to the digital twins (Whittle *et al.*, 2022 ▸).

## 5. Outlook and discussion

**Figure 8:**
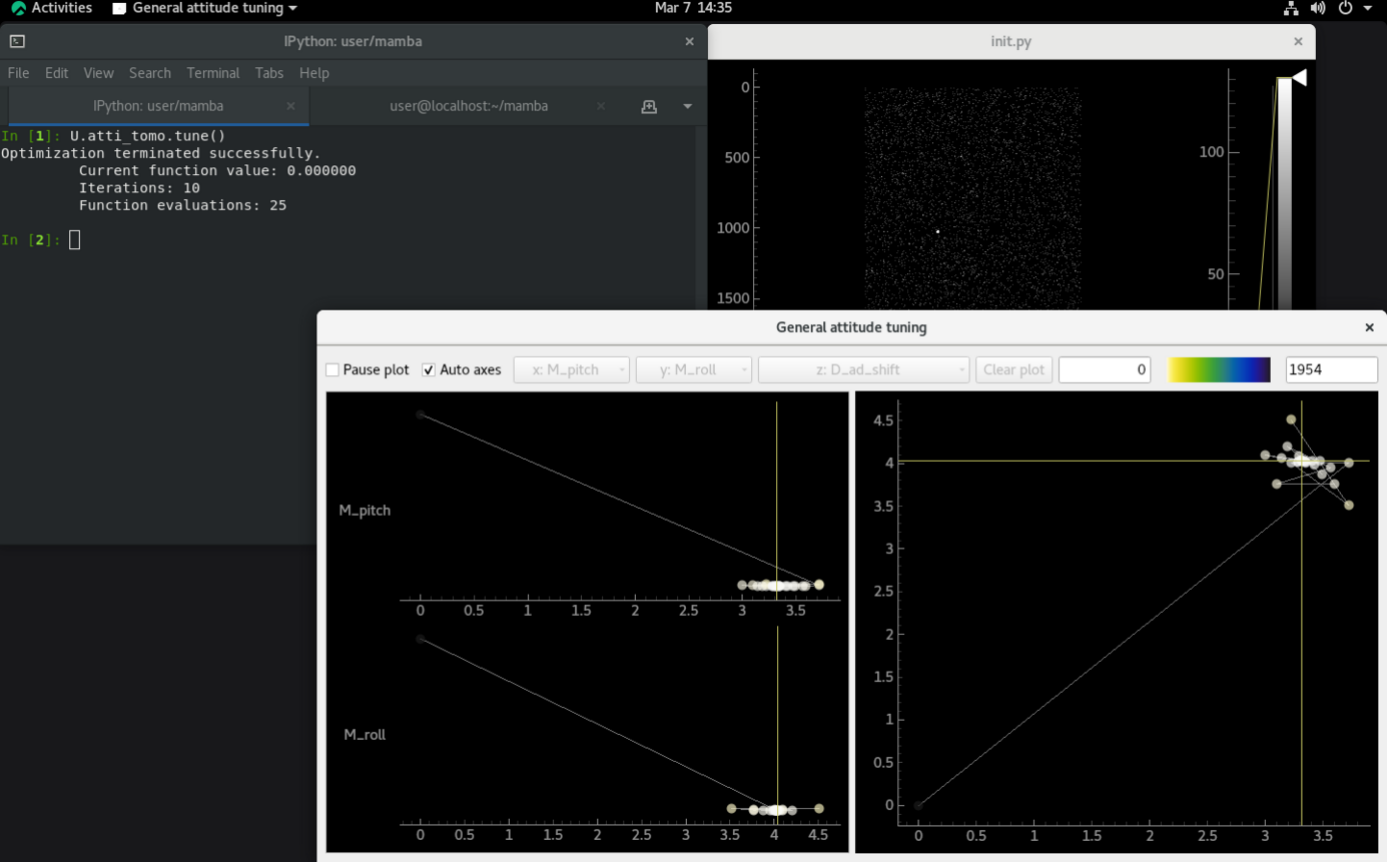
Alignment scheme used by Zhang *et al.* (2023*a* ▸) for the rotation (yaw) axis in tomography, reimplemented in our framework and running with a virtual beamline, where the detector images acquired during tomographic scans are monitored in the window in the upper right. To align the rotation axis precisely perpendicular to the beam direction, a strongly absorbing particle is added to a calibration sample, and its projection can be seen from the detector images. The projection’s trajectory during a tomographic scan with a non-optimal attitude (for the pitch and roll axes) is an ellipse or a diagonal line, which gradually degenerates into a horizontal line as the attitude improves. The tuning procedure is composed of a coarse move computed from the initial shape of the trajectory according to formulae available in the paper above, and then fine tuning of the attitude based on numerical optimization of the shape.

The examples in Sections 3 and 4 have demonstrated the use of complex evaluation functions and customized optimization algorithms in our attitude-tuning framework. Among the complexities summarized in Section 1, the only one not yet responded to in this paper is the use of objective parameters derived from scans. Our example for that is a reimplementation of the alignment scheme of Zhang *et al.* (2023*a* ▸) for the rotation axis in tomography (Fig. 8 ▸), whose code is given in the files 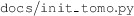 and 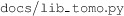 from the open-source edition of *Mamba*. As can be seen from 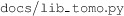, with the *MambaPlanner* mechanism (Li *et al.*, 2023*b* ▸), simple step scans are encapsulated under easy-to-use interfaces like 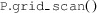, and their data processing pipeline can also be easily customized, *e.g.* for the handling of high-throughput data. More complex scans can also be done in similar ways, *e.g.* regular grid fly scans with 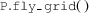, which can be very helpful in speeding up attitude tuning based on *e.g.* ptychography or tomography. With the examples above, and noticing the architectural versatility of our attitude-tuning framework (Section 2), we believe this framework is able to cover most attitude-tuning needs in a simple and maintainable way, especially during the normal operation of facilities/beamlines. On the other hand, we are also exploring the use of our framework during the construction phase of facilities/beamlines, where the relations between attitude parameters and objective parameters would often be much more complicated. We are also aware of requirements outside of beamlines that structurally resemble attitude tuning, *e.g.* the tuning of accelerators (Emery *et al.*, 2021 ▸; Whittle *et al.*, 2022 ▸) and the calibration of detectors (see the *xspress3-autocalib* program for the Xspress3 readout system for silicon drift detectors and high-purity germanium detectors); collaboration toward these directions has also been envisioned.

In the rest of this section, we would like to discuss a few issues we have encountered with current numerical optimization libraries during their application in attitude tuning. These issues originate from physical factors that are typically less frequently considered in the field of numerical optimization, and we hope the following discussions can raise mathematicians’ awareness of them. One of them concerns the serial nature of numerical optimization in attitude tuning: except for the tuning of digital twins, attitude tuning is based on manipulation of motor-like devices and readings from detectors, which are in general not susceptible to parallelization. Therefore acceleration strategies based on computational parallelization of optimization algorithms, like genetic algorithms, particle swarm optimization *etc.*, would often be unhelpful in attitude tuning. Furthermore, as the efficiency of attitude tuning often depends not only on the number of optimization moves, but also on the trajectory length due to the movement of motors, optimization algorithms that aim to shorten the trajectory may be a research direction worthy of systematic consideration. We are glad to learn that Bayesian optimization may take sampling expense (*e.g.* the movement of motors) into account, and is also resistant to the noise issue to be covered below. We hope other algorithms with similar advantages, especially those not ML-based, can also be explored. We additionally note that fly scans may be worth special attention in this research direction, since they can quickly sample large numbers of points on the motion trajectories.

**Figure 9:**
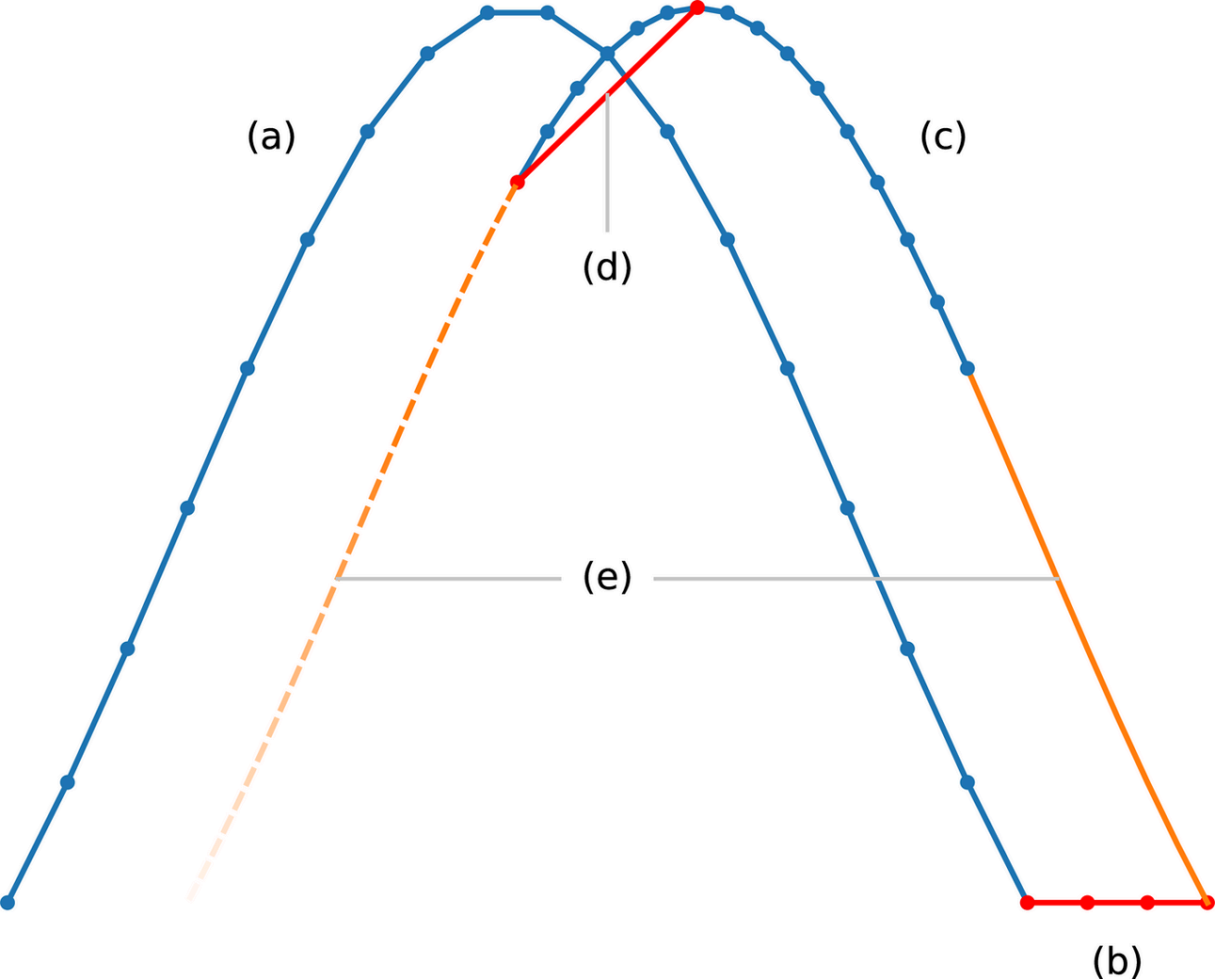
Objective parameter curves of the 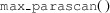 algorithm for each motor, where dots indicate scan points: (*a*) normal part of the coarse scan; (*b*) part of the coarse scan that may appear when the motor is stalled by a stopper; (*c*) the fine scan, which starts at the rightmost point where the objective value rises above a first threshold in the coarse scan, and stops when the objective value falls below a second threshold; (*d*) the final move to the peak position, deemed by the algorithm as the centre of the interval in the fine scan where the objective values were above the second threshold; (*e*) intervals skipped by the fine scan to save time.

Another issue we find is the precision limits in manipulation and measurement of physical systems, *e.g.* the step sizes of stepping motors. They can lead to pathological behaviours of some algorithms under certain conditions, which require workarounds like 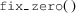 in the file 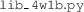 (Section 3). On a deeper level, this is because of the assumption in optimization algorithms that the readback values of position are always equal to the setpoints. Similarly, most optimization algorithms do not consider possible measurement errors in the objective value, except for *e.g.* those in the *Noisyopt* library (Mayer, 2016 ▸). Moreover, depending on the evaluation function (*e.g.* the one in 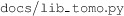), the objective value can have a lot of small plateaux, which may even occur with noise at the same time. A major type of algorithm that suffers from the three issues above is gradient-based optimization, which works badly in *e.g.* the 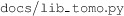 case above: in comparison with the Nelder–Mead algorithm, when 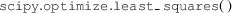 is used, the motors often jump too far, and the convergence is more often slower than on par or faster. In comparison with the minor noise levels in attitude parameters and objective parameters that stem from precision limits and measurement errors, hysteresis-like effects (*e.g.* motor backlashes) and drifting of physical systems (*e.g.* beam orbit drifting in accelerators and the decay-mode operations of storage rings; see Section 3 for the latter) can result in bigger problems, and may require special treatment in the optimization algorithms used. For instance, for multiple engineering reasons, the XRS spectrometer in Section 4 has no limit switches or motor encoders, and instead only has stopper blocks at the boundaries. So after a focusing motor becomes stalled by a stopper, the effect in Fig. 9 ▸(*b*) will be observed on the objective parameter. Consequently, the 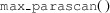 algorithm in Section 4 was designed with resistance against this effect in mind, and this resistance can be tuned with its threshold parameters.

## 6. Conclusion

The preparation steps in beamline experiments can be of particular interest in terms of automation, and a representative category in these steps is attitude tuning, including beam focusing, sample alignment *etc*. We find attitude tuning a ubiquitous requirement at light sources, and most of these requirements are peak finding in essence. Noting the nature of advanced light sources and the complexity of requirements at new light sources like HEPS, we have created a versatile framework for attitude tuning based on *Mamba*. We treat attitude tuning as a matter of numerical optimization, so based on the elements of numerical optimization and physical measurement, we implemented the 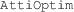 class which cooperates with *Bluesky*’s interfaces for motors and detectors, as well as optimization libraries like 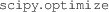. Aside from simple peak finding, by customizing 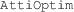 it is also possible to manipulate general motor-like devices, and to achieve effects like using the results from a scan as the raw data for each position. With help from *Mamba*’s infrastructure, ML/AI technologies can also be easily integrated into our attitude-tuning framework.

The first real-world example for our framework is the attitude tuning of the polycapillary lens on beamline 4W1B at BSRF, which demonstrates how to do simple peak finding with straightforward processing/evaluation functions. Also introduced with this example is a general-purpose visualization GUI for attitude tuning, and the support for multi-objective tuning in our framework. The next example is the tuning of a von Hamos XES spectrometer on 4W1B at BSRF, which uses a much more complex evaluation function. It also shows a way that human-in-the-loop control can be integrated into attitude tuning, where the human inputs are reused in normal ‘counting’ after the tuning. The final example is the tuning of the XRS spectrometer on B5 at HEPS, where the ‘optimization algorithm’ interface is ‘abused’ to do parallelized peak finding of multiple objective parameters, as well as the automated assignment of X-ray spots to analysers which is not numerical optimization at all.

With these examples, and noting the architectural versatility of our framework, we believe it is able to cover most attitude-tuning needs in a simple and maintainable way. Also reported is a virtual-beamline mechanism based on easily customisable simulated detectors and motors, which facilitates both testing for developers and training for users, as well as the encapsulation of digital twins. We note a few algorithmic issues in attitude tuning, which stem from physical factors less commonly considered in the field of numerical optimization, and which may require attention from mathematicians: the unsuitability of acceleration strategies based on computational parallelization; the importance of shortening the trajectory of optimization; the relevance of fly scans; the difference between readback values and setpoints; measurement errors and small plateaux in objective parameters; hysteresis-like effects; and drifting of physical systems.

## Supplementary Material

Code fragments. DOI: 10.1107/S1600577525003960/yn5119sup1.zip
